# Euglycemic Diabetic Ketoacidosis With Prolonged Glucosuria Associated With the Sodium-Glucose Cotransporter-2 Canagliflozin

**DOI:** 10.1177/2324709617712736

**Published:** 2017-06-08

**Authors:** Daniel A. Kelmenson, Kelsey Burr, Yusra Azhar, Paul Reynolds, Chelsea A. Baker, Neda Rasouli

**Affiliations:** 1 University of Colorado, Aurora, CO, USA

**Keywords:** canagliflozin, euglycemic diabetic ketoacidosis, glucosuria, Invokana, SGLT2 inhibitors

## Abstract

Sodium-glucose cotransporter-2 (SGLT2) inhibitors improve glycemic control by a reversible inhibition of the sodium-glucose cotransporters in the renal proximal tubules resulting in increased urinary glucose. This unique mechanism, independent of insulin secretion and beta cell function, has made this class of medication desirable in patients with type 2 diabetes. However in May 2015, the US Food and Drug Administration issued a safety warning pertaining to the development of diabetic ketoacidosis (DKA) with the use of SGLT2 inhibitors. DKA associated with SGLT2 inhibitors frequently develops in the absence of hyperglycemia, which makes the diagnosis more challenging. Due to the reversible inhibition of SGLT2 by this class of medication, a quick recovery of glucosuria after cessation of medication is expected. In this article, we present a case of a 50-year-old woman with type 2 diabetes who developed euglycemic DKA after initiating therapy with canagliflozin. This case of DKA associated with SGLT2 inhibitor use was unique due to her hypoglycemic presentation and persistent glucosuria. SGLT2 inhibitors such as canagliflozin may predispose patients not only to diabetic ketoacidosis but also to prolonged glucosuria.

## Introduction

Sodium-glucose cotransporter-2 (SGLT2) inhibitors are the newest agents that have been approved for the treatment of type 2 diabetes. SGLT2 is a sodium-dependent glucose transporter protein located in the proximal tubules of the kidneys, responsible for reabsorbing approximately 90% of the filtered glucose by the kidney.^1^ The increased filtered glucose to the kidneys in patients with type 2 diabetes causes an increased expression of SGLT2 protein in the proximal tubules, thereby resulting in increased glucose reabsorption and hyperglycemia.^2,3^ SGLT2 inhibitors improve glycemic control by reversible inhibition of SGLT2, resulting in decreased glucose reabsorption and increased glucose excretion in urine. SGLT2 inhibitor-induced glucosuria is proportional to the amount of glucose filtered by the kidneys; therefore, decreased plasma glucose concentration diminishes the action of SGLT2 inhibitors on the kidneys.^4,5^

In March 2013, the US Food and Drug Administration (FDA) approved the first SGLT2 inhibitor canagliflozin followed by the approval of dapagliflozin and empagliflozin in January 2014 and August 2014, respectively. SGLT2 inhibitors are recommended in combination with other antidiabetic medications or as monotherapy in patients who have contraindications or intolerance to metformin.^6^ One of the concerns with the use of SGLT2 inhibitors is an increased risk of diabetic ketoacidosis (DKA) with lower-than-anticipated glucose levels.^7-11^ It has been proposed that SGLT2 inhibitors increase lipid oxidation and glucagon production resulting in activation of the ketosis pathway, especially in patients with low insulin levels.^9^ Factors such as low caloric and fluid intake, concurrent illness, and alcohol use could also trigger ketosis.^7^

Due to the reversible inhibition of SGLT2 by this class of medication, a quick recovery of glucosuria is expected after cessation of the medication. In this article, we report a case of a patient treated with canagliflozin who presented with DKA, hypoglycemia, and unexpected prolonged glucosuria after cessation of canagliflozin, making the management of DKA more challenging.

## Case Report

A 50-year-old Caucasian woman presented with 4 days of nausea, vomiting, abdominal pain, and decreased oral intake. She had a 13-year history of type 2 diabetes complicated by gastroparesis. She was previously treated with insulin for gestational diabetes. After the diagnosis of type 2 diabetes, she was managed with metformin and sitagliptin. Due to an elevated hemoglobin A1c of 11.2%, canagliflozin 300 mg daily was added to her regimen 6 days prior to presentation. Her physical examination was notable for tachypnea (respiratory rate 28 bpm) and mild, diffuse tenderness to palpation on abdominal exam. She was overweight with a body mass index of 28. Labs revealed a creatinine of 0.54 mg/dL (estimated glomerular filtration rate >60 mL/min/1.73 m^2^), bicarbonate of 6 mmol/L, anion gap of 21, β-hydroxybutyrate of 90 mg/dL, glucose of 68 mg/dL, arterial pCO_2_ of 12 mm Hg, and arterial pH of 7.11. Urinalysis contained glucose >500 mg/dL and ketones 80 mg/dL.

**Figure 1.**
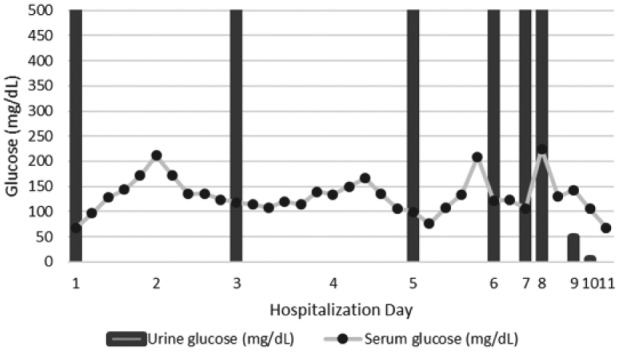
Serum glucose and urine glucose from day of admission.

The patient received 9 liters of normal saline with dextrose in the intensive care unit for suspected starvation ketoacidosis with no improvement in her bicarbonate level, anion gap, or acidosis. An insulin drip was added to her dextrose infusion with subsequent normalization of her acid-base status and resolution of ketonuria. Her nausea, vomiting, and abdominal pain also resolved and she was able to start eating. The insulin drip was stopped when the patient transferred from the intensive care unit to the floor where she subsequently experienced a recurrence of her abdominal pain and anion gap metabolic acidosis. These all resolved with reinstitution of the insulin drip, which was later bridged to subcutaneous insulin. After reinstituting insulin, her pH was 7.4, bicarbonate was 24 mmol/L, and anion gap was 8. She had significant glucosuria (>500 mg/dL) in the absence of hyperglycemia until day 9 of her hospitalization despite discontinuation of canagliflozin at admission (Figure 1). Glucosuria was present prior to dextrose therapy and persisted after dextrose was discontinued. The patient had no evidence for loss of other substances in the urine that would suggest other disorders such as Fanconi syndrome. She was discharged on basal subcutaneous insulin, and all other diabetic medications were stopped. At a follow-up visit 4 months later, she had an A1C of 6.4% without hypoglycemia on insulin glargine and no recurrence of DKA.

## Discussion

Canagliflozin increases urinary glucose excretion by lowering the renal threshold for glucose excretion in a dose-dependent manner with a maximal duration of effect of 24 hours for 300 mg and 12 hours for 100 mg, respectively.^12^ Since approval of canagliflozin, several cases of DKA in patients with type 1 or type 2 diabetes treated with SGLT2 inhibitors have been reported.^8^ A meta-analysis of canagliflozin trials reported the incidence of DKA as 0.52, 0.76, and 0.24 per 1000 patient-years for canagliflozin 100 mg, 300 mg, and a non-canagliflozin comparator, respectively.^13^ As a result of this, the FDA revised the labels to include warnings and precautions about the risks of ketoacidosis with SGLT2 inhibitors.^8^ Due to excretion of glucose in urine by SGLT2 inhibitors, most episodes of DKA reported with SGLT2 inhibitors are associated with a mild or moderate increase in blood glucose levels.^7-11^ In this case, DKA was associated with a low blood glucose of 68. Her DKA was likely triggered by low calorie intake due to severe gastroparesis, worsened by calorie loss through excretion of glucose in urine. This case is unique among recent reports of euglycemic DKA because glucosuria persisted for several days after stopping canagliflozin resulting in a negative calorie balance in the presence of low food intake.

Canagliflozin is a reversible and selective SGLT2 inhibitor.^12^ When given orally, canagliflozin reaches its peak plasma concentration in 1 to 2 hours and a steady state in 4 days with minimal accumulation after successive redosing.^12^ Canagliflozin is primarily excreted in the urine (~33%) and feces (~60%).^12^ The half-life for orally administered 100 mg and 300 mg of canagliflozin is 10.6 and 13.1 hours, respectively.^12^ Therefore, the SGLT2 inhibition should disappear 2 to 3 days after discontinuation of canagliflozin,^12,14^ resulting in increased renal reabsorption of glucose and less glucosuria. This patient continued to have urine glucose >500 mg/dL for 9 days after stopping canagliflozin despite normal kidney function. Her urine glucose was disproportionately high for blood glucose levels (Figure 1). During the period of prolonged glucosuria along with her continued poor oral intake, she had recurrent mild ketonemia despite continuing insulin treatment.

Persistent renal glucosuria has been reported in people with SGLT2 gene mutations,^15,16^ but our patient’s glucosuria resolved after 10 days, suggesting that the effect was due to inhibition of the transporters by canagliflozin. More recently, a case report describes the increase in glomerular filtration rate and administration of exogenous insulin as the possible mechanisms in enhancing the effects of SGLT2 inhibitors on the kidneys in promoting glucosuria.^17^ Canagliflozin is primarily metabolized by uridine diphosphate glycosyltransferase (UGT) enzymes.^12^ UGT polymorphisms, which are well described in the literature, could be implicated in the prolonged drug effect we observed.^18^

## Conclusion

The diagnosis of DKA in patients treated with SGLT2 inhibitors may initially be overlooked in the absence of hyperglycemia. Clinicians should be aware of the potential risk of euglycemic DKA associated with SGLT2 inhibitors so that early management of this serious but treatable condition can commence. As our report suggests, the SGLT2 inhibitor effect can persist for several days (up to 10 days) after discontinuation, further complicating the management of DKA in these patients.
